# Focused ultrasound robotic system for very small bore magnetic resonance imaging

**DOI:** 10.1002/rcs.2165

**Published:** 2020-09-22

**Authors:** Christakis Damianou, Marinos Giannakou, Nikolas Evripidou, Stefan Kegel, Peter Huber, Juergen Jenne

**Affiliations:** ^1^ Electrical Engineering and Computer Engineering and Informatics Department Cyprus University of Technology Limassol Cyprus; ^2^ MEDSONIC LTD Limassol Cyprus; ^3^ German Cancer Research Center (DKFZ) Heidelberg Germany; ^4^ MEDIRI GmbH Heidelberg Germany

**Keywords:** HIFU, MRgFUS, MRI, positioning, robotic system

## Abstract

**Background:**

A magnetic resonance imaging (MRI) compatible robotic system for focused ultrasound was developed for small animal like mice or rats that fits into a 9.4 T MRI scanner (Bruker Biospec 9420, Bruker Biospin, Ettlingen, Germany). The robotic system includes two computer‐controlled linear stages.

**Materials and Methods:**

The robotic system was evaluated in a mouse‐shaped, real‐size agar‐based mimicking material, which has similar acoustical properties as soft tissues. The agar content was 6% weight per volume (w/v), 4% w/v silica while the rest was degassed water. The transducer used has a diameter of 4 cm, operates with 2.6 MHz and focuses energy at 5 cm.

**Results:**

The MRI compatibility of the robotic system was evaluated in a 9.4 T small animal scanner. The efficacy of the ultrasonic transducer was evaluated in the mimicking material using temperature measurements.

**Conclusions:**

The proposed robotic system can be utilized in a 9.4 T small animal MRI scanner. The proposed system is functional, compact and simple thus providing a useful tool for preclinical research in mice and rats.

## 1 INTRODUCTION

The technology of magnetic resonance guided focused ultrasound (MRgFUS) is directed recently mostly for brain applications after the major success of treating essential tremor non‐invasively.^1^ Another promising application for brain is the treatment of glioblastoma using MRgFUS.^2^ Because of these two major and promising applications, a lot of efforts have been directed towards research regarding brain. In the last decade, a lot of emphasis was given in opening the blood brain barrier (BBB) in order to push therapeutic drugs in targets in the brain. Recently, MRgFUS was explored in treating Alzheimer by opening the BBB.^3^ Experimental studies for BBB disruption using focused ultrasound started in 2001 by Hynynen et al.^4^ by sonicating the brain of 18 rabbits. The most popular animal for experimentation is the mouse^5^, ^6^, ^7^, ^8^, ^9^ because it provides useful data with minimal cost and effort.

Therefore, there is significant demand for exploring new concepts in the area of MRgFUS. The exploration of new concepts requires robotic systems for mice. One of the first positioning devices was reported by the team of Chopra^10^ that developed an magnetic resonance imaging (MRI)‐compatible three‐axis robotic FUS system for small animals.

The company MEDSONIC developed several robotic systems for animal use. One such system was developed for use in the rabbit brain.^11^, ^12^ Other systems were dedicated for preclinical use for prostate,^13^, ^14^ gynaecological applications,^15^ and small animals.^16^ The French company Image Guided Therapy (Pessac, France)^17^ developed also an MRgFUS system for small animal experiments using phased arrays, a rather complicated and expensive solution for small animals.

The main goal in this article was to develop an MRgFUS robotic system for mice that operates in the environment of a 9.4 T scanner. The proposed system is reduced in size in order to fit in a 9.4 T preclinical system. With the use of a standard resonators coil, the bore diameter of MRI is reduced to 7 cm. The MRI‐compatibility of the materials used (transducer, motors, plastics, encoders and screws) was evaluated extensively in other studies reported by this group^11^, ^12^, ^13^, ^14^, ^15^ and therefore such results are not presented in this article.

The proposed robotic system can accommodate an ultrasonic transducer with 4 cm diameter. Based on the size of the robot and size of mice, the maximum radius of curvature that can be accommodated is 5 cm. Due to the fact that mice are small in size, there is no need for stage in the X axis of the MRI. Therefore two linear axes are needed (Z and Y in MRI planes). The advantage of using the proposed system over the phased array (electronic steering of the focus) is that the focus is steered mechanically making the system less complex and thus inexpensive. Since mice are small, it is possible to achieve transcranial ablation with a single element transducer.

## 2 MATERIALS AND METHODS

### 2.1 Robotic system

**FIGURE 1:**
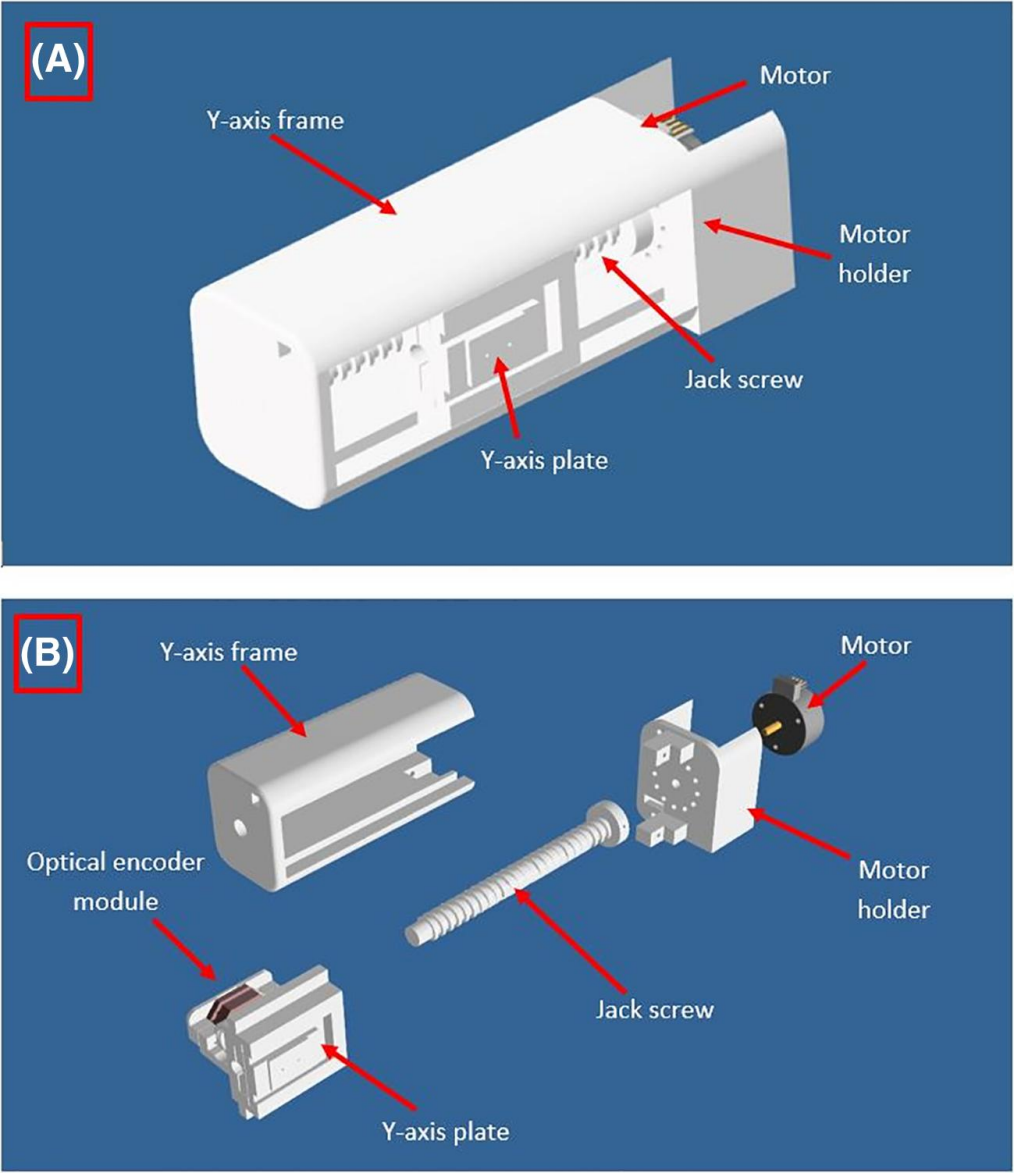
(A) Drawing of the linear axis for motion along the magnetic resonance imaging Y axis. (B) Exploded view diagram showing the linear axis components

**FIGURE 2:**
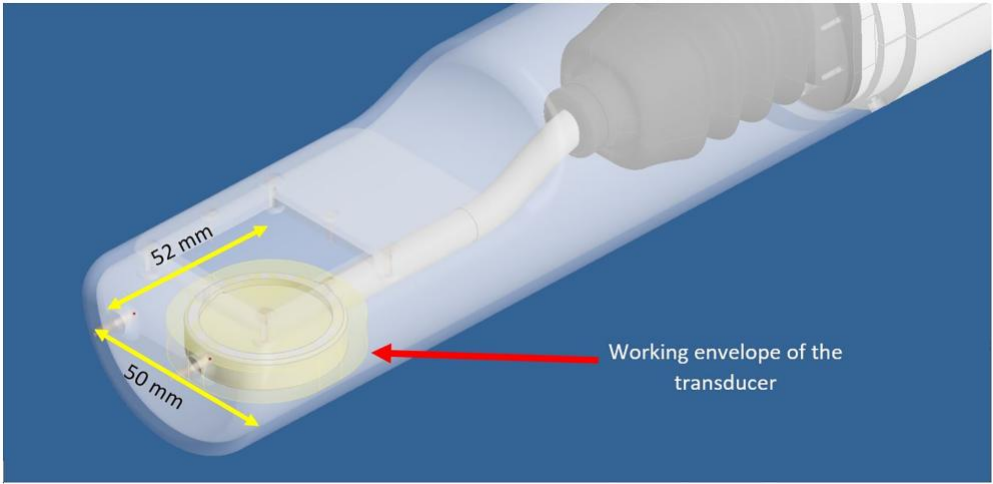
Front part of the robotic system

The robotic system includes two computer‐controlled axes (Z and Y in MRI). Figure 1 shows the drawing of the linear actuator for motion along the MRI Y axis. All the parts of the robotic system were 3D printed and made out of the MR compatible plastic material Acrylonitrile Butadiene Styrene (ABS). A moving plate was coupled to a threaded plastic screw which was attached to the shaft of an ultrasonic motor (USR 30‐S3, Shinsei Kogyo Corp.). With this arrangement the shaft converts the angular motion to linear. An encoder strip of transparent Mylar film (with 500 lines per inch) was placed in the Y‐axis frame and moves inside the encoder module (US Digital Corporation) which is not visible in this drawing. For both axes, the encoder module EM1‐0‐500‐I (US Digital Corporation) was used. The encoder output is connected to the counter input of a data acquisition board USB 6251 (National instruments). The robotic system was designed using the software Microstation (V8, Bentley Systems, Inc.) and manufactured with a 3D printer (FDM400, Stratasys) using ABS material. The Z‐axis design was identical to the design of the Y axis. Figure 2 shows the front part of the robotic system (the working envelope of the transducer is highlighted in yellow). In this part, the small animal is placed in prone position (natural position). It is possible to place the animal in supine position by using a specially designed holder. The volume of the working envelope was calculated by the outer diameter of the transducer (22 mm), the motion range of 2.8 × 3 cm in Y‐ and Z‐axis respectively and the thickness of the transducer (10 mm). The robotic system weights around 2.2 Kg. The flow rate of the degassed water was 10 mL/s. The circulating water was kept at room temperature.

**FIGURE 3:**
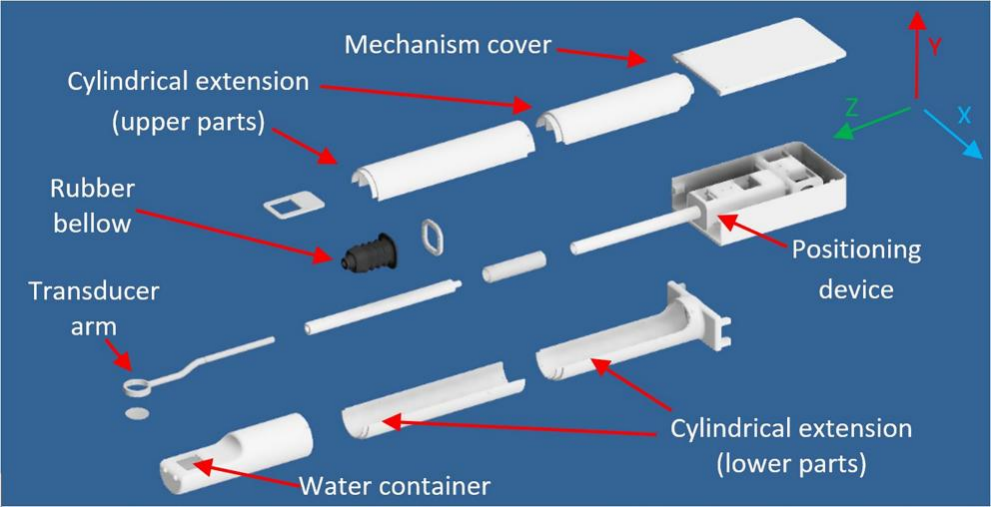
Interior of the robotic system highlighting the two linear axes, the transducer arm, ultrasonic transducer and water space

Figure 3 shows the interior of the robotic system highlighting the two linear axes, the transducer arm, ultrasonic transducer and water space. The transducer arm goes through a plastic bellow which isolates the water space from the mechanical part of the two axes. Transducer was immersed in a container which was filled with degassed water.

Compared to previous small animal positioning devices developed by our group,^16^ the main difference was the usability at 9.4 T and the size of the motor which is almost 50% smaller. With these smaller size motors, we were able to develop more compact robotic systems.

**FIGURE 4:**
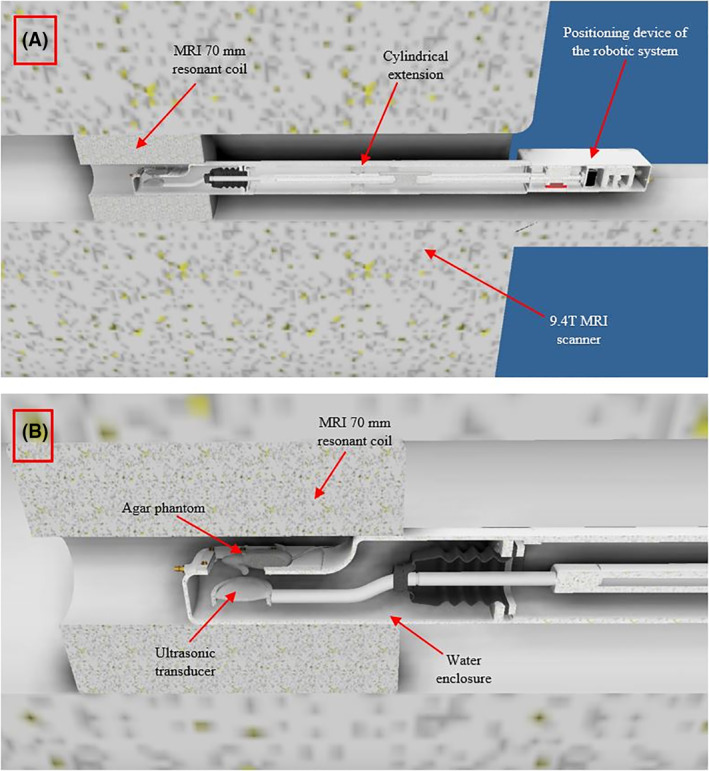
(A) Drawing of the robot placed in the 9.4 T magnetic resonance imaging. (B) A closer view of the water enclosure with the mouse model placed on the acoustic opening

**FIGURE 5:**
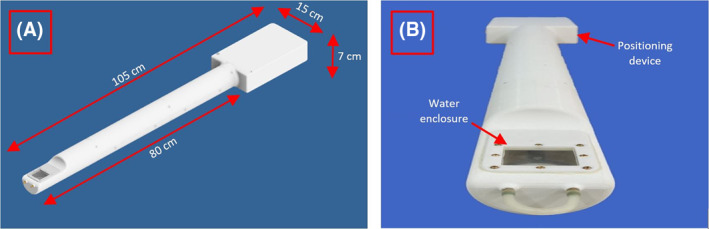
(A) Drawing of the robotic system. (B) Photo of the developed robotic system

The extended arm of the robotic system is cylindrical with an external diameter of 70 mm and can be placed inside the standard 70 mm resonator of the 9.4 T MRI scanner. The Robotic system has a maximum height of 7 cm, a length of 105 cm and a width of 15 cm. The cylindrical extension that connects the positioning device with the water enclosure, has a diameter of 7 cm and it is approximately 80 cm in length. Figure 4A shows the placement of the device inside the 9.4 T MRI scanner. Figure 4B shows a closer view of the water enclosure's drawing with the mouse model placed on the acoustic opening. Figure 5A shows the drawing of the robotic system, and Figure 5B shows the photo of the developed robotic system.

### 2.2 FUS system

The FUS system included of a signal generator (HP 33120A, Agilent Technologies), an RF amplifier (AG1012, T & C Power Conversion, Inc.). The ultrasonic transducer was made out of P762‐type piezoceramic (Ferroperm). A backing material (epoxy) withstanding 150°C was used as a backing material for the transducer element. The transducer's impedance was matched to 50 Ω. To reduce the transmission of high frequency harmonics, a custom made low‐pass filter was used (10 MHz cut‐off frequency). The proton frequency of the 9.4 T MRI scanner is at 400 MHz. The transducer used has a diameter of 4 cm, operates with 2.6 MHz and focuses energy at 5 cm.

### 2.3 Software

A user‐friendly program was developed written in C # (Visual Studio 2010 Express, Microsoft Corporation). The software controls the motion of the positioning device and has the following functionalities: (a) Interfacing with MRI and (b) automatic or manual motion of the two axes. Automatic motion is achieved by six different algorithms.^18^ (c) MR thermometry and (d) ultrasound control (frequency, power, sonication time, duty factor and pulse duration).

**FIGURE 6:**
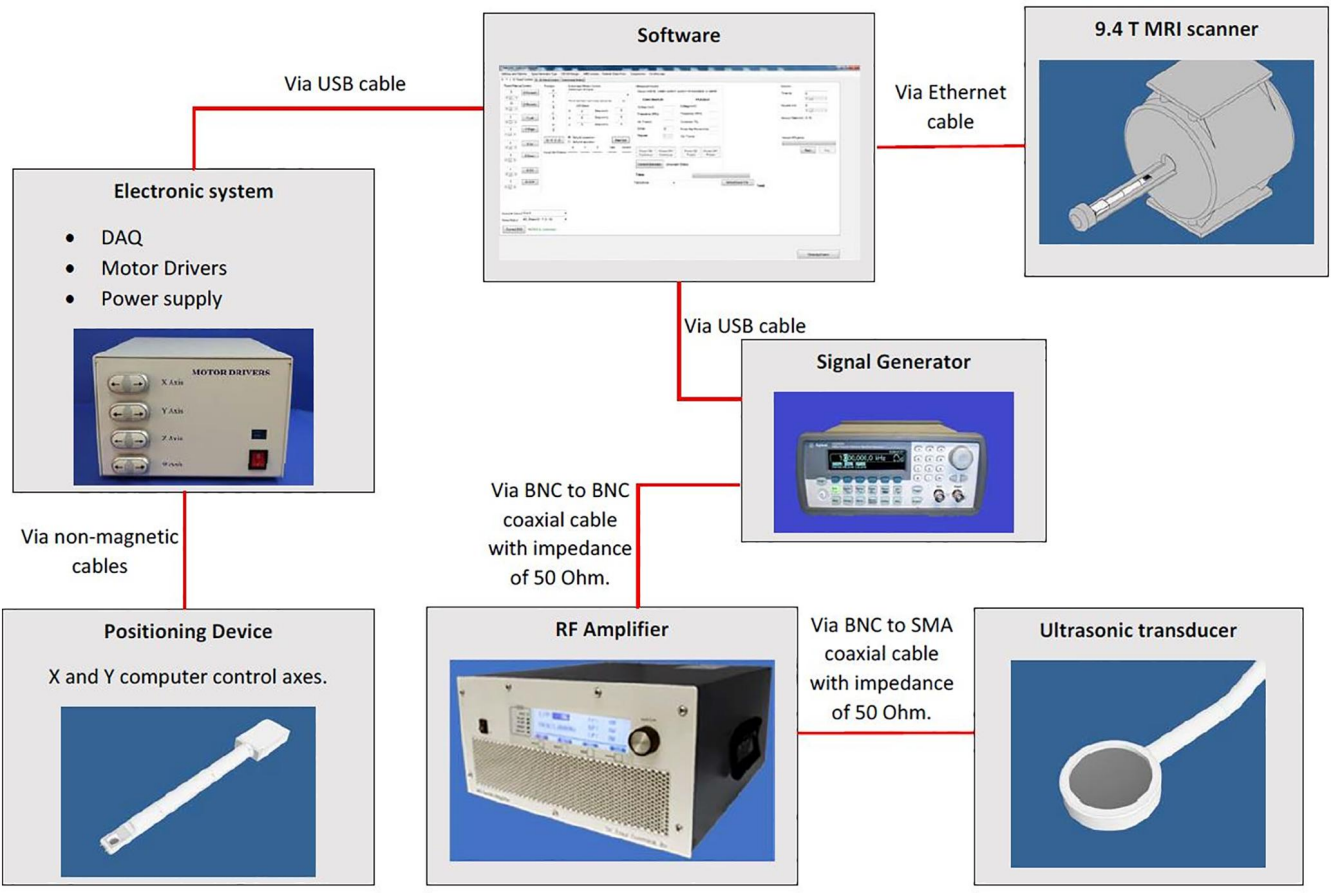
Workflow diagram of the software

Figure 6 shows the device network of the computer connected to the 9.4 T MRI scanner, the robotic system and the high intensity focused ultrasound (HIFU) system. The software acquires the encoder pulses via the data acquisition board which is part of the electronic system. The accuracy of the device was achieved with the use of optical encoders that have a resolution of 500 lines per inch. In a manual motion, the desired distance and direction must be defined. For the automatic motion, a grid pattern must be set by selecting the number of steps, the distance on each direction and time between steps.

After every sonication, the heat conduction increases the temperature of the nearby tissue. A delay between each step can be added in the software to avoid the near field heating effect. Six different algorithms were developed to produce non‐linear motion patterns to avoid sonications between adjacent targets to reduce the delay between steps, thus reducing the treatment time.

### 2.4 Electronic system

The electronic system includes a DC supply (24 V, 2 A) which drives the piezoelectric Shinsei motors. Cables connect the motor drivers and a data acquisition interface card (USB 6251, NI) via a connecting block. The USB 6251 data acquisition card includes timing and digital I/O modules. The ultrasonic motors were driven when the ground and clockwise terminals of the motor drivers were connected to the same potential (clockwise rotation) or when the ground and anti‐clockwise terminals of the motor drivers were connected to the same potential (anti‐clockwise rotation). Accuracy of the stages was tested extensively in other robotic systems that have identical robots and encoders.^11^, ^12^, ^13^, ^14^, ^15^ The use of MR compatible encoders established a positional error of the linear stages in the order of 0.1 mm.

### 2.5 Agar‐based mimicking material

The functionality of the transducer and the MRI compatibility of the robot was evaluated in an agar‐based mimicking material. The mixture of the agar‐based mimicking material consisted of 6% weight per volume (w/v) agar, 4% w/v silica dioxide and the rest were degassed water. This agar‐based mimicking material was already used successfully in other studies.^19^, ^20^ This phantom was used to measure the temperature produced by the system's transducer. Additionally, the phantom was used during the MR compatibility of the robotic system as the imaging load. The shape and size of the phantom is very close to real mice. Specifically, the phantom has a length of 78 mm, width of 28 mm and height 26 mm. This was achieved by producing with 3D printer, a specially designed mould of a real mouse. The volume required to fill the mould was approximately 27 ml. The attenuation of the phantom was found to be equal to 1.1 dB/cm‐MHz.^20^

### 2.6 Magnetic resonance imaging

The robotic system was tested in a 9.4 T MR system with PET insert (Bruker Biospec 9420, Bruker Biospin). The BRUKER Biospin coil (RF RES, 1H) was used which is tuned at a resonance frequency 400 MHz and has an internal diameter of 72 mm. High‐resolution MRI was performed to visualize the robot/mimicking material/transducer arrangement. Thus a FLASH‐sequence (Fast Low Angle Shot) was used with the following parameters: repetition time TR = 15 ms, echo time TE = 3 ms, flip angle FA = 10°, matrix 128 × 128, slice thickness = 1 mm and field of view FOV = 90 × 90 mm to show possible field distortions and influence to the MRI.

## 3 RESULTS

**FIGURE 7:**
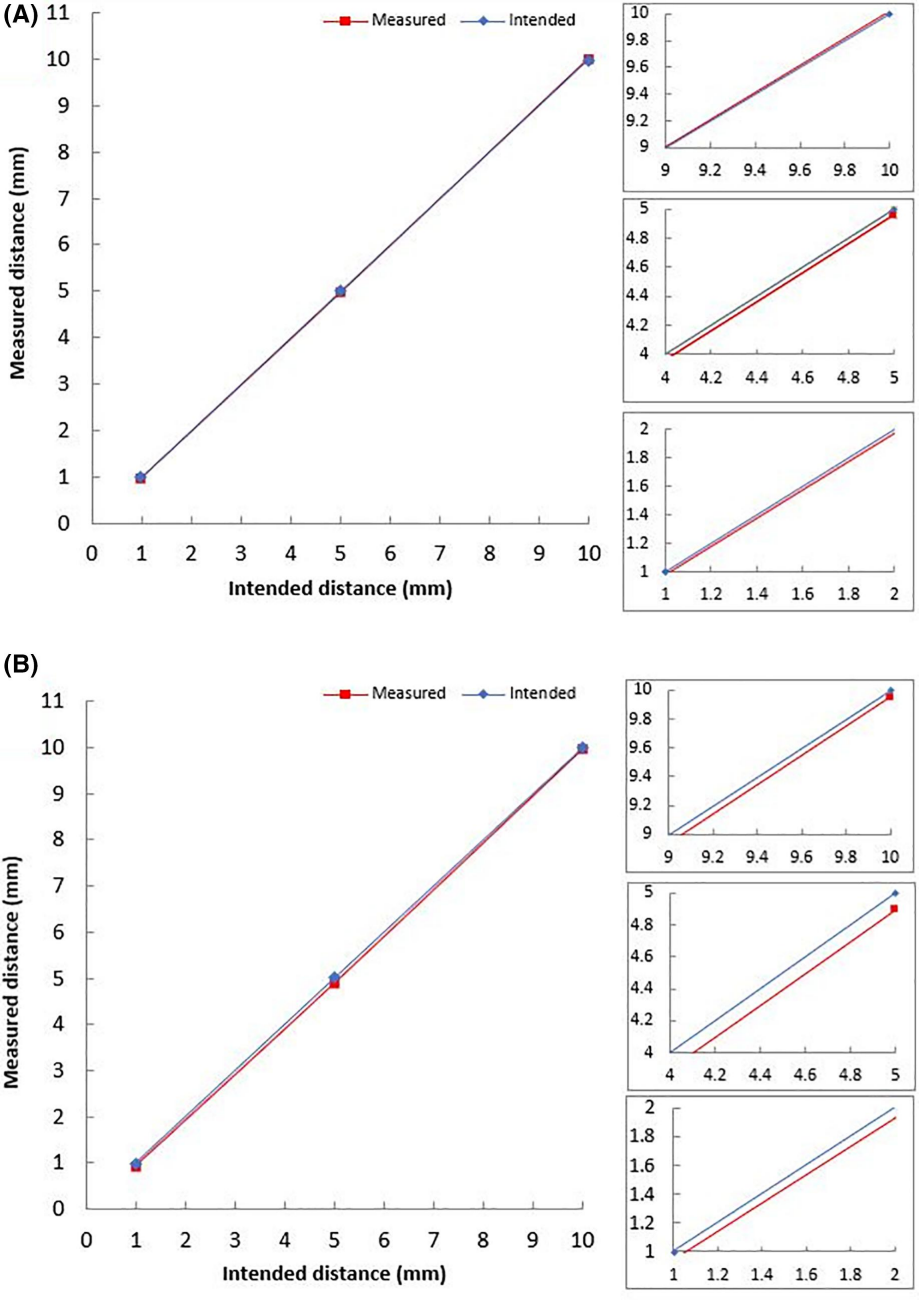
(A) Measured distance versus intended distance for the Z axis forward motion (left) and zoomed areas of the graph (right). (B) Measured distance versus intended distance for the Y axis forward motion (left) and zoomed areas of the graph (right)

Figure 7A shows the difference of intended step (blue line) to actual distance measured (red line) on the Z axis. The blue line represents the ideal expected motion and the red line represents what was actually achieved. Therefore, the difference between the two represents the motion error. Figure 7B shows the same graph for the Y axis. Motion steps of 1, 5 and 10 mm were evaluated. The average error measured for 20 movements of 1 mm was 0.02 mm, for 5 mm was 0.0375 mm and for 10 mm was 0.024 mm. The step of 1 mm is typically used in high‐frequency sonications and represents the minimum step that is normally used. The 5 mm step is used typically with low‐frequency sonications or with high‐power sonications. The 10 mm step is typically used to place the robot in home position.

**FIGURE 8:**
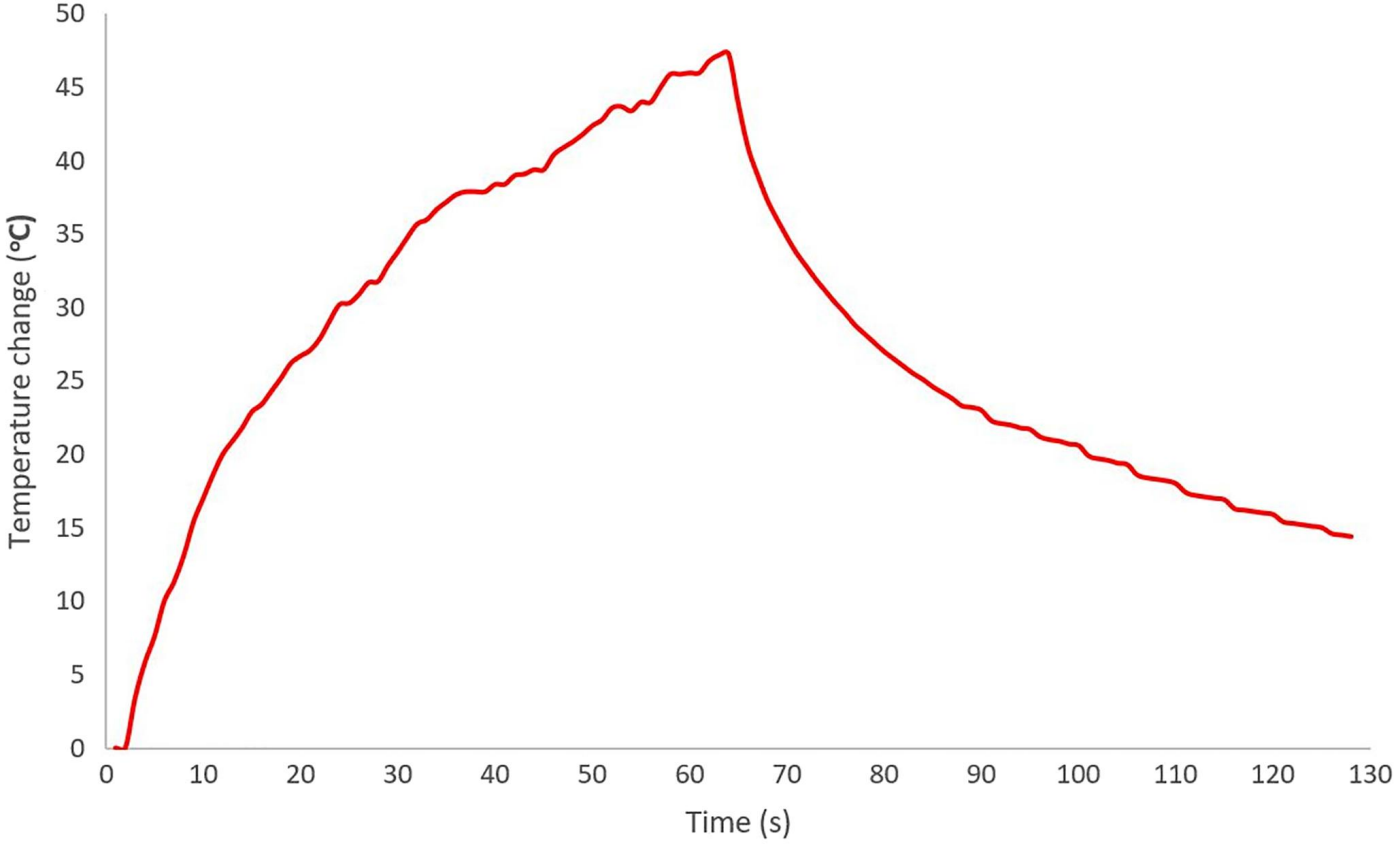
Temperature versus time in the agar‐based phantom using acoustical power of 15 W. The transducer used has a diameter of 4 cm, operates with 2.6 MHz and focuses energy at 5 cm

The ability of the transducer to create high temperature was tested in the laboratory setting using the agar‐based phantom. Figure 8 shows the temperature versus time in the agar‐based phantom using acoustical power of 15 W and the temperature sensor placed directly at the focal spot. Note that a temperature change of 47°C was produced in approximately 60 s. Similar temperatures are observed in tissue with the same transducer and sonication parameters. The temperature increases due to the ultrasonic power, and when temperature increases the rate of temperature decreases due to conduction. When the ultrasound is turn OFF, then the temperature drops rapidly.

**FIGURE 9:**
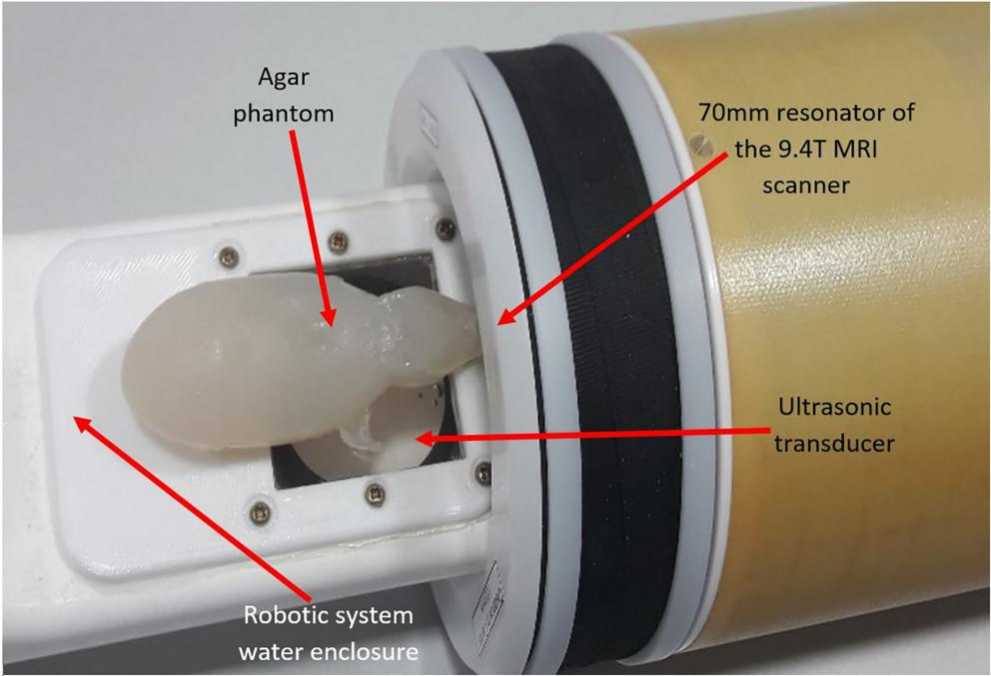
Placement of the mimicking mouse in the front part of the robotic system

**FIGURE 10:**
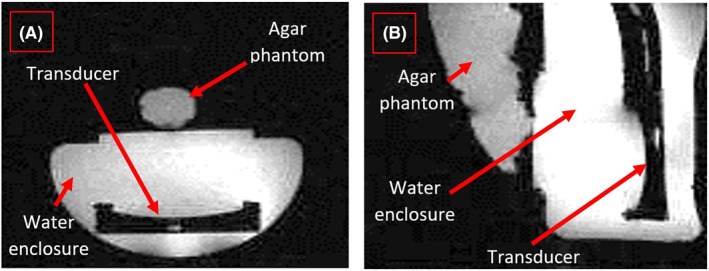
(A) Coronal image in the 9.4 T magnetic resonance imaging (MRI) scanner highlighting the transducer, water enclosure and mouse‐mimicking material. (B) Axial image in the 9.4 T MRI. The transducer used has a diameter of 4 cm, operates with 2.6 MHz and focuses energy at 5 cm

Figure 9 shows the placement of the mimicking mouse in the front part of the robotic system. The robotic system is then inserted in the coil of the 9.4 T MRI scanner. Water was poured on the membrane film in order to provide good coupling between the robot and mouse‐mimicking material. Figure 10A shows the coronal image in the 9.4 T MRI scanner highlighting the transducer, water enclosure and mouse‐mimicking material. Figure 10B shows the axial image in the 9.4 T MRI scanner highlighting the transducer, water enclosure and mouse‐mimicking material. Note that no major artefacts were caused due to the presence of the ultrasonic transducer. Some artefacts that appeared were due to the transducer cable and are located outside of the clinical MRI area of interest.

## 4 DISCUSSION

An MRgFUS positioning device was developed that can be used with mice and rats in a commercial 9.4 T small animal MRI scanner with a bore diameter of 7 cm. Due to the size of the animal only two axes are needed (called Z and Y in this article in compliance with the MRI axes). The robotic system can be navigated in two axes with a good spatial accuracy. This range of motion is more than enough for the size of mice or rats. The MRI compatibility of the system was tested successfully in a 9.4 T MRI system, using high‐resolution imaging. In another article, we will present extensive MR thermometry of ultrasonic ablation.

In this robotic system, the animal is placed in supine position, but with a special holder it can be placed also in prone position. The main innovation of this device is that its size is small compared to the available devices. Currently, the only device to perform this^17^ uses phased arrays which moves the beam electronically. The proposed robotic system uses a single element transducer, which makes the system simple and affordable and yet as effective as the other available systems. In our opinion for preclinical efforts in mice, the use of a robotic system with two axes and the use of a single element transducer can result into a functional system. In this preliminary design, a mouse‐mimicking material was used based on water and agar. In a follow up study, we will use real mice or rats to acquire data for this robotic system. With these small animals, it will be possible to explore fully an attractive application (e.g., BBB using HIFU).

## CONFLICT OF INTEREST

All authors declare no conflict of interest.

## Supporting information

Supplementary Material

## References

[1] Elias K , Huss D , Voss T , et al. A pilot study of focused ultrasound thalamotomy for essential tremor. N Engl J Med. 2013;369(7):640‐648. 23944301 10.1056/NEJMoa1300962

[2] Coluccia D , Fandino J , Schwyzer L , et al. First noninvasive thermal ablation of a brain tumor with MR‐guided focused ultrasound. J Ther Ultrasound. 2014;2(1):1‐7. 25671132 10.1186/2050-5736-2-17 PMC4322509

[3] Meng Y , MacIntosh BJ , Shirzadi Z , et al. Resting state functional connectivity changes after MR‐guided focused ultrasound mediated blood‐brain barrier opening in patients with Alzheimer's disease. Neuroimage. 2019;200:275‐280. 31254646 10.1016/j.neuroimage.2019.06.060

[4] Hynynen K , McDannold N , Vykhodtseva N , Jolesz FA . Noninvasive MR imaging‐guided focal opening of the blood‐brain barrier in rabbits. Radiology. 2001;220(3):640‐646. 11526261 10.1148/radiol.2202001804

[5] Rapoport N , Payne A , Dillon C , et al. Focused ultrasound‐mediated drug delivery to pancreatic cancer in a mouse model. J Ther Ultrasound. 2013;1(1):11. 25516800 10.1186/2050-5736-1-11 PMC4265944

[6] Chen X , Griffin R , Webber J , et al. WE‐E‐220‐04: focused ultrasound ablation of tumour hypoxic tissue of small animals under PET and MRI guidance. Med Phys. 2011;38(6):3824.

[7] Choi JJ , Pernot M , Small SA , Konofagou EE . Noninvasive blood‐brain barrier opening in live mice. In AIP Conference Proceedings. 2006;829:271‐275.

[8] Choi JJ , Small SA , Konofagou EE . Optimization of blood‐brain barrier opening in mice using focused ultrasound. In Proceedings–IEEE Ultrasonics Symposium. 2006;1:540‐543.

[9] Choi JJ , Wang S , Morrison B , Konofagou EE . Focused ultrasound‐induced molecular delivery through the blood‐brain barrier. In Proceedings–IEEE Ultrasonics Symposium. 2007:1192‐1195.

[10] Chopra R , Curiel L , Staruch R , et al. An MRI‐compatible system for focused ultrasound experiments in small animal models. Med Phys. 2009;36(5):1867‐1874. 19544806 10.1118/1.3115680 PMC2736709

[11] Damianou C , Ioannides K , Milonas N . Positioning device for MRI‐guided high intensity focused ultrasound system. Int J Med Robot Comput Assist Surg. 2008;2(6):335‐345.

[12] Mylonas N , Damianou C . MR compatible positioning device for guiding a focused ultrasound system for the treatment of brain deseases. Int J Med Robot Comput Assist Surg. 2013;10(1):1‐10. 10.1002/rcs.1501 23744569

[13] Yiallouras C , Mylonas N , Damianou C . MR compatible positioning device for guiding a Focused ultrasound system for transrectal treatment of prostate cancer. Int J Comput Ass Rad. 2014;9(4):745‐753. 10.1007/s11548-013-0964-x 24337790

[14] Yiallouras C , Ioannides C , Dadakova T , et al. Three axis MR conditional robot for high intensity focused ultrasound for treating prostate diseases. J Ther Ultrasound. 2015;3(1):2. 25657846 10.1186/s40349-014-0023-2 PMC4318438

[15] Epaminonda E , Drakos T , Kalogirou C , et al. MRI guided focused ultrasound robotic system for the treatment of gynaecological tumors. Int J Med Robot. 2016;12(1):46‐52. 25808561 10.1002/rcs.1653

[16] Yiannakou M , Menikou G , Yiallouras C , et al. MRI guided focused ultrasound robotic system for animal experiments. Int J Med Robot Comput Assist Surg. 2017;13(4):e1804. 10.1002/rcs.1804 28211622

[17] Image Guided Therapy . 2020 http://www.imageguidedtherapy.com/. Accessed May 1, 2020.

[18] Yiannakou M , Trimikliniotis M , Yiallouras C , Damianou C . Evaluation of focused ultrasound algorithms: issues for reducing pre‐focal heating and treatment time. Ultrasonics. 2016;65:145‐153. 26476464 10.1016/j.ultras.2015.10.007

[19] Menikou G , Dadakova T , Pavlina M , et al. MRI compatible head phantom for ultrasound surgery. Ultrasonics. 2015;57:144‐152. 25482534 10.1016/j.ultras.2014.11.004

[20] Menikou G , Damianou C . Acoustic and thermal characterization of agar based phantoms used for evaluating focused ultrasound exposures. J Ther Ultrasound. 2017;5(1):14. 28572977 10.1186/s40349-017-0093-z PMC5452295

