# Supplementary material for: Focused ultrasound robotic system for very small bore magnetic resonance imaging
Source: Int J Med Robot. 2020 Sep 22;16(6):e2165. doi: 10.1002/rcs.2165 (PMC7816236; doi:10.1002/rcs.2165)
Supplement: Supplementary file 1 — Supplementary Material [file RCS-16-e2165-s001.docx]

Reviewing: 1

Comments to the Author Most of suggestions have been addressed. But some problems still exist in the revised manuscript.

1. Authors said more results were added in the experimental results, but it’s still too confused. Fig.7 is unclear. Why measured distance and intended distance are chosen as horizontal and vertical coordinates? Why draw two lines? Three points (1 mm, 5 mm and 10 mm) is quite a small sample.

We have added text to better explain why fig. 7 is important. We explained why we measured 1, 5 and 20 mm steps. Note that for each step 20 measurements were performed.

2. Fig.4, Fig.5 and Fig.6 in the Page 16 of 31 should be changed to Fig.8, Fig.9 and Fig.10 respectively.

It is strange because in our word file numbering appears correctly, but not in the pdf. Maybe this was caused by the fact that we use links for figures. Anyway this appears correctly now.

3. The format of the references is still disordered and not uniform, such as some unabbreviated Journal names and the number of authors’ names.

Now corrected.

Reviewing: 2

Comments to the Author. This is an interesting, overall well-written manuscript on the enabling technologies for robot assisted focused ultrasound. The paper is ready for publication.

Thanks, no action here.
